# Correction: Prevalence and correlates of psychological distress among diabetes mellitus adults in the Jilin province in China: a cross-sectional study

**DOI:** 10.7717/peerj.2869/correction-1

**Published:** 2017-12-07

**Authors:** Shuang Qiu, Hongxuan Sun, Yawen Liu, Joseph Sam Kanu, Ri Li, Yaqin Yu, Xufeng Huang, Bo Li, Xiangyang Zhang

**Affiliations:** 1 Department of Epidemiology and Biostatistics, School of Public Health, Jilin University, Changchun, China; 2 Illawarra Health and Medical Research Institute, School of Medicine, University of Wollongong, Wollongong, Australia; 3 Psychiatry Research Center, Beijing Hui-Long-Guan Hospital, Peking University, Beijing, China

Corrigendum:

After publication, the authors noticed that five authors’ names were listed incorrectly. The author list and author contributions have been amended so that: Xuan Hong Sun is now listed as Hongxuan Sun. Wen Ya Liu is now listed as Yawen Liu. Qin Ya Yu is now listed as Yaqin Yu. Feng Xu Huang is now listed as Xufeng Huang. Yang Xiang Zhang is now listed as Xiangyang Zhang.

