# Supplementary material for: Prevalence and correlates of psychological distress among diabetes mellitus adults in the Jilin province in China: a cross-sectional study
Source: PeerJ. 2017 Jan 17;5:e2869. doi: 10.7717/peerj.2869 (PMC5244878; doi:10.7717/peerj.2869)
Supplement: Data S1 [file peerj-05-2869-s001.zip › raw data/The code of raw data csv.docx]

| Variables | Code | Code | Code | Code | Code |
| --- | --- | --- | --- | --- | --- |
| Community | 0=“rural” | 1=“urban” |  |  |  |
| AGE (continuous) |  |  |  |  |  |
| Race | 0=“Han nationality” | 1=“Non Han minority” | |  |  |
| Marriage | 0=“Married” | 1=“Never married” | 2=“Divorce or separate” | 3=Widowed | |
| Occupation | 0=“Others” | 1=“Mental worker” | 2=“Manual work” | |  |
| AGE3G | 0=“18~” | 1=“45~” | 2=“60-79” |  |  |
| GHQscore (continuous) |  |  |  |  |  |
| Distress | 0=“No” | 1=“Yes” |  |  |  |
| AwarenessDM | 0=“No” | 1=“Yes” |  |  |  |
| BMI | 0=“＜24” | 1=“≥24” |  |  |  |
| DailySleepDuration | 0=“≥7” | 1=“＜7” |  |  |  |
| NumberOfOtherIllnesses | 1=“≥3” | 2=“2” | 3=“1” | 4=“0” |  |
| FamlilyHistoryOfDM | 0=“No” | 1=“Yes” |  |  |  |
| EducationalLevel | 0=“Primary school and below” | 1=“Junior high school” | 2=“Senior high/technical secondary school and above” | | |
| FamilyAverageMonthlyIncome | 1=“< 500” | 2=“500~1000” | 3=“1000~2000” | 4=“2000~3000” | 5=“≥3000” |
| gender | 0=“male” | 1=“female” |  |  |  |
