# Supplementary material for: Prevalence and correlates of psychological distress among diabetes mellitus adults in the Jilin province in China: a cross-sectional study
Source: PeerJ. 2017 Jan 17;5:e2869. doi: 10.7717/peerj.2869 (PMC5244878; doi:10.7717/peerj.2869)
Supplement: Data S1 [file peerj-05-2869-s001.zip › raw data/Notes of raw data.docx]

Dear editor：

In order to make it convenient for you to open the files, I have uploaded two files named "raw data", the filetypes of the two files are "csv" and "sav" which can be opened by EXCEL and SPSS software respectively. I have clearly illustrated the meaning of each code in the word file named “The code of raw data”.

Thank you very much.

Kind regards

Bo Li
